# Supplementary material for: Sadness regulation strategies and measurement: A scoping review
Source: PLoS One. 2021 Aug 13;16(8):e0256088. doi: 10.1371/journal.pone.0256088 (PMC8362967; doi:10.1371/journal.pone.0256088)
Supplement: Attachment — Submitted filename: Response to Reviewers.docx [file pone.0256088.s002.docx]

**Response to Reviewers’ comments**

Name of journal: PLOSONE

Manuscript NO: PONE-D-20-37486

Manuscript title: **Sadness regulation strategies and measurement: A Scoping review.**

Dear Editor,

Thank you very much for your kind e-mail, which gave us the opportunity to revise our manuscript. We edited the paper according to the reviewer’s comments. Please find enclosed response letter. Each comment has been answered accordingly in the manuscript and each text that has been amended was written in red colour and highlighted in Gray in the revised manuscript. We hope that the revised version will fulfil the requirements for publication in PLOS ONE. Thank you for your consideration. We look forward to hearing from you.

Your sincerely,

Sumaia Mohammed Zaid, Ph.D. candidate

Department of Educational psychology and counselling

Faculty of Education,

University of Malaya

Malaysia.

**Response to Reviewers’ comments**

First of all, we want to deeply thank both the Editor and the Reviewer 1 for their valuable time that they gave to read our manuscript and for their constructive feedback and valuable comments that guided us to improve our manuscript quality. The present manuscript aimed to explore sadness regulation strategies that were reported in the literature and the available measures of sadness regulation. Second, we change the type of the manuscript from **systematic review to scoping review;** therefore, the title was changed accordingly as the content and aim fit more into scoping reviews papers. Herein, we wrote each comment and our response to it.

**Response to editor comments:**

1- The abstract should contain the essential findings only. Technical or statistical details should not be reported in the abstract. I suggest reworking the abstract in the revised version of the manuscript.

**Response:**

Thank you for your comment, we rewrote the abstract and remove the statistical details.

2- I agree with reviewer 1 that the structure of the introduction needs to be reworked. At first, I suggest providing a brief overview about the goals of the study. In the next step, the state of the art in the field should be portrayed and relevant empirical work should be referred here. Then, the "problem" or the "gap" in the knowledge should be stated, and the hypotheses or research questions should result from this reasoning. Since you are particularly focused on methodological aspects of empirical studies, I would suggest my own work that could potentially serve as a tutorial to guide you in the process of revision. Although my work deals with a different topic (sustained attention), it could nevertheless help you by providing a showcase of how to configure empirical work in a method-oriented review.

**Response:**

Thank you for your constructive comment, we rewrote the introduction, we tried to provide more information about emotion regulation model and explained the scope of the study and the gap in the literature.

3- The method of aggregating studies lacks important information. It is important not to miss existing studies so I suggest taking all efforts to carefully screen the body of literature in the revision process. Also, the goals of this review should be distinguished from other existing review articles on the same or similar topic.

**Response:**

We aimed to explore the knowledge gab in sadness regulation concept in general. Therefore, we used scoping review and our key words was focused on the term sadness regulation specifically. We amid to explore the strategies that are used to regulate sadness as well as to explore the available measures of sadness regulation. Furthermore, the researchers aimed to mapping sadness regulation as integrated unite. We attempted to add key words such as “strategies” and “measurement” but the results were more into economic field.

4- While I appreciate qualitative reviews, it is important to find ways of condensing the relevant information. The outcome of the review should be informative, only stating that studies are methodologically different, using different design methods, and so on, is not sufficient as it does not inform the reader about the results of empirical work in the field.

**Response:**

Thank you for your comment, we highlighted key findings for all the articles that were reviewed, a column was added to table (1) that we dedicated to present the key findings kindly check the last column in Table 1.

5- Tables should be presented according to APA standards. Oversize tables should be avoided, if possible. I suggest reconsidering the information presented in tables in the revision of the manuscript.

**Response:**

Thank you for your comment, we fix the format of the table and modified its content.

6- The final outcome should be condensed in a take home message. My suggestion is to try answering the following questions during the revision process: What are the key knowledge aggregated from the many empirical studies? What are the gaps that should be addressed in the future? What are the critical points that could be due to the use of different designs or other technical aspects of previous studies? What are the guidelines that could be provided to improve future research based on this review?

**Response:**

Thank you for your helpful comment, we tried to highlight these points in discussion part, we outlined the discussion in subheadings namely, sadness regulation strategies, sadness regulation measurement, summary of methodological aspects, challenges, and recommendation of previous works that we reviewed. Kindly check discussion part.

7- The manuscript should be checked for grammar errors and typos.

**Response:**

Thank you for your comment, we sent for proofreading.

**Response to reviewer comments**

Abstract
- As the first sentence stands for now, it may seem like the authors juxtapose depression and sadness. Please revise.

**Response:**

Thank you for your comment, we rewrote the abstract and remove the first sentence related to depression.

Introduction
- Generally, the introduction is too short and lacks a thorough theoretical and clinical presentation of central concepts which leads to research questions.
The concepts are not described in a nuanced fashion. It lacks to mention previous reviews about emotion regulation strategies, and I miss several central references (e.g. the very much cited Aldao et al., 2010; Webb et al, 2012, Augustine & Hemenover, 2009). The introduction also lacks a theoretical “anchor”: here it is natural to describe and cite Gross’ updated process model of emotion regulation (2015).

**Response:**

Thank you for your informative comment, we rewrote the introduction we included the Gross model 2015 as well as previous reviews.

- Again, it may seem like the authors juxtapose depression and sadness. I would omit the word depression, and start the introduction about sadness (from sentence two; “Sadness is a basic human emotion…”., and describe that some people have difficulties regulating these normal feelings, and consequently develop depression.

**Response:**

Thank you for your comment, we deleted the first sentence and started introduction “Sadness is a basic human emotion…”.

- Line 60-61: This sentence does not make sense. Please revise/clarify

**Response:**

Thank you for your comment, there was extra coma separated the sentence and make the meaning discontinues, we deleted the coma and modify the sentence so the meaning becomes clearer. “Individuals face many challenges when coping with negative emotions including sadness throughout their lives [6, 7]. Therefore, the capability to….” Kindly check line 52-54.

- Line 63-: “Suppression is a type of…”. The authors claim that suppression is a nonadaptive “method”, but they should be more nuanced in this claim, as suppression sometimes is adaptive (dependent on context and culture).

**Response:**

Thank you for your comment, we completely agree with your point. Here we meant that in the context of sadness, suppression is considered as non-adaptive strategy because “it has been found that using expression suppression is ineffectual in reducing sadness experience (Gross & John, 2003; Schindler & Querengässer, 2018)”. Accordingly, we modify the sentence to become “Suppression is a type of non-adaptive method of emotion regulation for negative emotions like sadness”

- I miss more about the adaptive features of sadness. In its present form, the introduction is a bit “negative” around sadness, although it, in fact is a very important feeling that in fact is adaptive (e.g. increased social support).

**Response:**

Thank you for your comment, we talked about the positive side of sadness kindly check lines 65-72.

Method
- Search keywords: I am concerned that the current keywords will not capture all of the literature available. The search terms should also include known emotion regulation strategies (e.g. rumination, suppression, savouring)

**Response:**

We aimed to explore the knowledge gab in sadness regulation concept in general. Therefore, we used scoping review and our key words was focused on the term sadness regulation specifically. We amid to explore the strategies that are used to regulate sadness as well as to explore the available measures of sadness regulation. Furthermore, the researchers aimed to mapping sadness regulation as integrated unite. We attempted to add key words such as “strategies” and “measurement” but the results were more into economic field.

Results
- Page 7, lines 152-154. I would not name “emotion regulation coping strategies” as an emotion regulation strategy.

- Line 160: I would not name “dysregulation expression strategy” as a strategy

**Response:**

Thank you for your comment, we change the term “strategy” to “aspect” as it is considered an aspect of sadness regulation. Most of the studies examined three aspects of sadness regulation which are: (1) tendency to mask or inhibit the expression of sadness (inhibition), (2) the expression of sadness in culturally unacceptable ways (dysregulation), and (3) managing sadness in adaptive ways (coping) (e.g., Cassano & Perry-Parrish, 2007).

- Page 8: “For more strategies, see table 1”: I would like the authors to describe these.

**Response:**

Thank you for your comment, we described more strategies that were reposted frequently in the previous studies and we presented the strategies in figure 2 (P.14).

Discussion
OK, but I miss the more thorough discussion in terms of the issues that lacks in the introduction.

**Response:**

Thank you for your comment, we outline the discussion in subheadings namely, sadness regulation strategies, sadness regulation measurement, summary of methodological aspects, challenges, and recommendation of previous works that we reviewed. Kindly check discussion part.
