# Supplementary material for: Sadness regulation strategies and measurement: A scoping review
Source: PLoS One. 2021 Aug 13;16(8):e0256088. doi: 10.1371/journal.pone.0256088 (PMC8362967; doi:10.1371/journal.pone.0256088)
Supplement: Attachment — Submitted filename: Response to Reviewers.docx [file pone.0256088.s003.docx]

**Response to Reviewers’ comments**

Name of journal: PLOS ONE

Manuscript NO: PONE-D-20-37486R1

Manuscript title: **Sadness regulation strategies and measurement: A Scoping review.**

Dear Editor,

Thank you very much for your e-mail, and we deeply apologize for making reviewers go for the second round of review. We revised the paper according to the reviewer’s comments. Please find the enclosed response letter. Each comment has been answered accordingly in the manuscript and each text that has been amended was written in red colour in the revised manuscript. We hope that the revised version will fulfil the requirements for publication in PLOS ONE. Thank you for your consideration. We look forward to hearing from you.

Yours sincerely,

Sumaia Mohammed Zaid, PhD candidate

Department of Educational psychology and counselling

Faculty of Education,

University of Malaya

Malaysia.

**Response to Reviewer’ comments**

First of all, we want to profoundly thank the Reviewers for their valuable time that they gave to read our manuscript and for their constructive feedback and valuable comments that guided us to improve our manuscript quality. The present manuscript aimed to explore sadness regulation strategies that were reported in the literature and the available measures of sadness regulation. Herein, we wrote each comment and our response to it.

**Abstract**
1- Abstract is better written. From reading the abstract, I expect that the authors wish to provide a descriptive overview of research that include strategies to regulate sadness, and an overview of the measures used in these research trials. However, the research questions also state that the authors want to examine psychometric properties with the measures. I would like the results of these properties also to be reported in the abstract.

**Response:**

Thanks for your comment, we included the psychometric properties results in the abstract. Researchers also modified the word examine in the research question to “to explore the existing instruments used to measure sadness regulation along with their psychometric properties”.

2- Sentence “while some of these studies used informant report…” does not make sense.

**Response:**

Thanks for your comment, we rewrote the sentence properly “ Moreover, we identified four types of measures namely self-reported, informant report (parents or peers), open-ended questions and emotion regulation instructions.”.

**Introduction**
Generally, the authors have provided new research and better language in this revision. However, the language is not good enough for publication in the current form.
The presentation of Gross’ model is sufficient but is somewhat not integrated with the text that follows. The examples of sadness strategies should be explained in terms of the Gross model (page 5).

**Response:**

Thanks for your comment, we tried to integrate the Gross model with the following paragraphs also to explain the strategies based on the Gross model.

**Method**
1- I appreciate that the authors have omitted the term “systematic review”.

**Response:**

We are sorry for forgetting to replace the word systematic review in the whole manuscript, we did check the whole text and replace the word systematic review with scoping review.

2- Fig2, prisma flow diagram: Please state the reasons for exclusion.

**Response:**

Thanks for your comment, we provided the reasons for exclusion in Fig 2.

**Results**
Again, I think the reporting is inconsistent, and the results also have major flaws regarding language.
1- I think the reporting of the results are somewhat unclear, referring to approximations, where I wonder about the exact number (e.g. p 9, “approximately 110 different strategies”; page 10, line 7 “reported in several studies”, etc.)

**Response:**

The sentence “approximately 110 different strategies” refers to the total number of the strategies that were found across the 40 articles included in the scoping review. The phrase “reported in several studies” refers to distraction strategy, we modify it to “The third common strategy is distraction (employed in 8 studies), which refers to cognitively and behaviourally removing oneself from negative emotions by engaging in activities unrelated to the present situation [e.g., 50, 52-54], Paez, Martinez-Sanchez, Mendiburo [55].”

2- P. 10: Wording “when people are unable to deal with their sadness and emotions, they tend to seek experts…”, sounds odd. People are dealing with their emotions by using strategies, such as seeking social support.

**Response:**

Thanks for your comment, what we mean by this sentence is that (People use seeking social support strategy to regulate their emotions especially when they are unable to deal with their negative emotions on their own, so they seek others intervention (e.g., experts, closed people) to help them overcome their negative emotions). We rewrote it “People seek social support (experts, closely related people) especially to regulate their negative emotions”.

3- Page 10: “[Rumination] was discussed in six studies”: odd wording, please revise.

**Response:**

Thanks for your comment, it was modified to “Rumination is the fifth common strategy employed in 6 of the studies. This strategy refers to the tendency of repeatedly thinking about the feelings along with their causes and consequences)”.

4- Fig 3: please use a table instead, sorting strategies by frequencies.

**Response:**

Thanks for your comment, we replaced the figure with a table, we presented each article and the strategies reported in that article.

5- Page 12-13: too long parentheses starting on the last line of page 12.

**Response:**

Thanks for your comment, we rewrote this part (p. 13-14).

6- Page 24: “this systematic review”: please revise to “scoping”

**Response:**

Thanks for your comment, we replace systematic review with scoping review in the whole text.

7- I like the idea of presenting psychometric properties of the included measures. However, I think the paper would increase its strength if the authors instead of describing these attributes, did a quality assessment of the included measures. This would inform the research field to a greater degree than in its current form. Although I think the authors have done a good job with describing and defining challenges within each study, it is a lot of information that is hard to systematize and process for the reader. Inclusion of a quality assessment would be more informative.

**Response:**

Thanks for your comment, the researchers aimed to explore and describe the strategies used to regulate sadness and the measures used to assess sadness regulation and highlight their psychometric properties. We did not aim to evaluate the psychometric properties of the existing measures. Researchers conducted a simple quality assessment for the included measures per study and instrument using COSMIN assessment of psychometric properties. Most of the studies included in this scoping review used pre-validated instruments and reported only Cronbach alpha. Kindly check the results section pages 27-34.

**Discussion**
The discussion also needs to be worked on in terms of language, and some of the sentences does not make sense.

1- The authors state on page 28 that expressive suppression was the most common strategy preferred. What study are the authors referring to? Further, it sounds odd that people suppress sadness to avoid hurting others and preserve harmonious relationships.

**Response:**

Thanks for your comment, we deleted the word preferred, and rewrote it this way, “we identified that expressive suppression was the most commonly used strategy across the reviewed articles. According to Huwaë and Schaafsma [79], people in collectivistic culture tend to suppress their negative or positive emotions to avoid hurting others and to preserve harmonious relationship”.

2- P 30: refer to the study as a scoping review

**Response:**

Thanks for your comment, we change it to scoping review.
