# Supplementary material for: Sadness regulation strategies and measurement: A scoping review
Source: PLoS One. 2021 Aug 13;16(8):e0256088. doi: 10.1371/journal.pone.0256088 (PMC8362967; doi:10.1371/journal.pone.0256088)
Supplement: Attachment — Submitted filename: Response to Reviewers.docx [file pone.0256088.s004.docx]

**Response to Reviewers’ comments**

Name of journal: PLOS ONE

Manuscript NO: PONE-D-20-37486R2

Manuscript title: **Sadness regulation strategies and measurement: A Scoping review.**

Dear Editor,

Thank you very much for your e-mail, and your encouraging evaluation. We revised the manuscript according to the reviewer’s comments. Please find the enclosed response letter. Each comment has been addressed accordingly in the manuscript and the amendments were written in red colour in the revised manuscript. We hope that the revised version will fulfil the requirements for publication in PLOS ONE. Thank you for your consideration. We are looking forward to hearing from you.

Yours sincerely,

Sumaia Mohammed Zaid, Ph.D. candidate

Department of Educational psychology and counselling

Faculty of Education,

University of Malaya

Malaysia.

**Response to Reviewer’ comments**

First of all, we want to profoundly thank the Reviewers for their valuable time given to read the second revised version of our manuscript and for their constructive feedback and valuable comments that guided us to improve our manuscript quality. The present manuscript aimed to explore sadness regulation strategies that were reported in the literature and the available measures of sadness regulation. Herein, we wrote each comment and our response to it.

- Language. I strongly recommend the authors to submit the manuscript to a copy-editing service to increase the quality of language. There are several parts of the manuscript that lack sufficient quality of the English language, especially in the abstract (p.2, lines 37-38; 40-43) and the introduction (e.g. p 3, lines 59-60; 69-71).

**Response:**

We have fixed the highlighted sentences in the abstract and introduction and then sent the manuscript to the proofreading and editing service for the third time.

- lack of essential information regarding quality assessment: I miss information about who did these assessments (one or two authors?). In case just one author did this, I on beforehand strongly recommend the authors to add another rater to increase validity and reliability of the assessment. Please also report how the authors reached consensus in cases of dissimilar ratings.

**Response:**

Thanks for your comment, we forgot to explain this point in the manuscript. Actually, two reviewers (SZ and ST) independently applied the COSMIN checklist to evaluate the methodological quality of the psychometric properties reported in the included studies. Any discrepancies between the two reviewers were resolved by involving a third reviewer who is an expert in psychometrics. We followed the same method of resolving discrepancies that was used by (Cartagena-Ramos, D., Fuentealba-Torres, M., Rebustini, F., Leite, A. C. A. B., de Andrade Alvarenga, W., Arcêncio, R. A., ... & Nascimento, L. C. (2018). Systematic review of the psychometric properties of instruments to measure sexual desire. *BMC medical research methodology*, *18*(1), 1-13)
